# Torsional Necrosis of an Output Loop Internal Hernia After Roux-en-Y Choledochojejunostomy: A Case Report

**DOI:** 10.3389/fsurg.2019.00051

**Published:** 2019-09-06

**Authors:** Chao Huang, Zhikun Ning, Zhengming Zhu

**Affiliations:** Department of Gastrointestinal Surgery, The Second Affiliated Hospital of Nanchang University, Nanchang, China

**Keywords:** internal hernia, volvulus, bowel resection, surgical operation, Roux-en-Y choledochojejunostomy

## Abstract

Ultra-short bowel syndrome (USBS) refers to a clinical condition characterized mainly by severe diarrhea and nutritional disorders caused by the residual small intestine length of <30 cm (1). After Roux-en-Y choledochojejunostomy, the output loop herniated from the back of the anti-reverse peristalsis loop, resulting in intestinal torsion and necrosis, and leading to this rare case of internal hernia. Patients with torsion often show severe abdominal pain and persistent aggravation, but this symptom lacks specificity, making it easy to be misdiagnosed. Acute intestinal torsion and necrosis often lead to large-scale small bowel resection, resulting in USBS. We report a case of torsional necrosis of an output loop internal hernia after Roux-en-Y choledochojejunostomy, providing certain reference for the diagnosis and management of such cases.

## Introduction

Since the jejunum was characterized by its ease of dissociation, its ability for anastomosis with other organs, and the forward peristalsis of the Y-shaped jejunum loop, Cesar Roux first used it in 1893 to reconstruct the digestive tract. In the early 1940s, the American surgeon Allen Whipple applied the Roux-en-Y anastomosis method to choledochojejunostomy. Today, the Roux-en-Y anastomosis of the bile duct to the jejunum remains one of the main methods of biliary reconstruction. Due to the maturity of this technology, its ease of operation, and good drainage effect, it has been widely employed in clinical practice, although cases of torsional necrosis of the output loop internal hernia are rarely reported. The twisting of the intestine easily forms a closed fistula, accompanied by a blood circulation disorder, causing a strangulated intestinal obstruction. Patients with a twisted intestine often show severe abdominal pain and persistent aggravation, but this symptom lacks specificity, meaning it is easily misdiagnosed. Torsional intestinal obstruction is a serious acute abdominal issue, and timely diagnosis is the key to treatment. Previous studies (2) have shown that with the increasing use of computed tomography (CT) in acute abdominal pain, the diagnostic accuracy of acute intestinal obstruction is significantly improved. Mastering the key signs of diagnosis and conducting rigorous analysis can significantly improve the accuracy of diagnosis and guide clinical treatment. Acute intestinal torsion and necrosis often lead to large-scale small bowel resection, resulting in the USBS, avoidance of which is the key to the success of the operation. We report a case of torsional necrosis of an output loop internal hernia after Roux-en-Y choledochojejunostomy, preoperatively diagnosed by abdominal CT and successful surgical treatment.

## Case Report

A 72-year-old woman was admitted to the hospital due to “persistent severe abdominal pain for one day.” In 2005, a Roux-en Y choledochojejunostomy had been performed for choledocholithiasis. After the operation, the patient successfully recovered and was discharged. After discharge, the patient did not have any discomfort. On December 13 2017, the patient, without obvious inducement, presented with persistent severe abdominal pain, nausea and vomiting, but no radiating pain, no fever, and no jaundice. She went to the local hospital for an abdominal CT examination, which suggested an intestinal obstruction, and intrahepatic bile duct stones. Treatment with antispasmodic, analgesic, anti-infection, and intravenous hydration did not significantly improve the symptoms. She was transferred to our hospital for treatment.

### Physical Examination

The blood pressure was 80/50 mmHg, the heart rate was 118 beats/min, the respiratory rate was 26 breaths /min and no yellow staining was found on the skin or sclera. The abdomen was protuberant and the right abdomen had a surgical scar ~10 cm long. The abdominal muscle was tense, and there was obvious right upper abdominal tenderness and rebound pain. Percussion gave a drum sound with shifting dullness. The bowel sounds were absent in auscultation. An abdominal puncture yielded a bloody fluid. Urgent examination of the chest and abdomen by a CT scan showed that the output loop was twisted and necrotic due to internal hernia; there was also ascites and a common bile duct stone.

**Figure 1:**
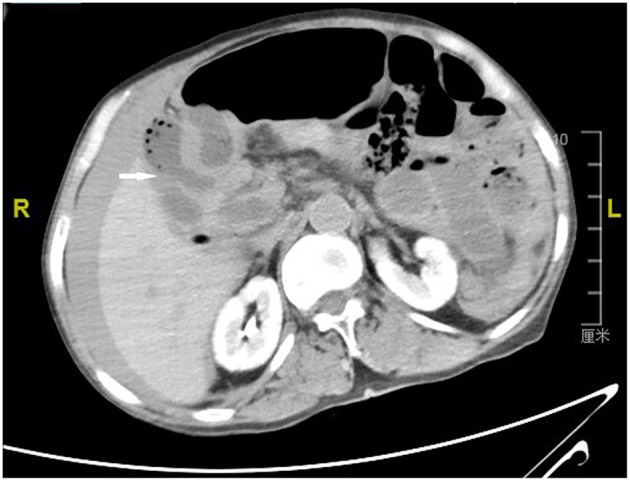
Computerized axial tomographic scan showing the original choledochojejunostomy (arrow).

**Figure 2:**
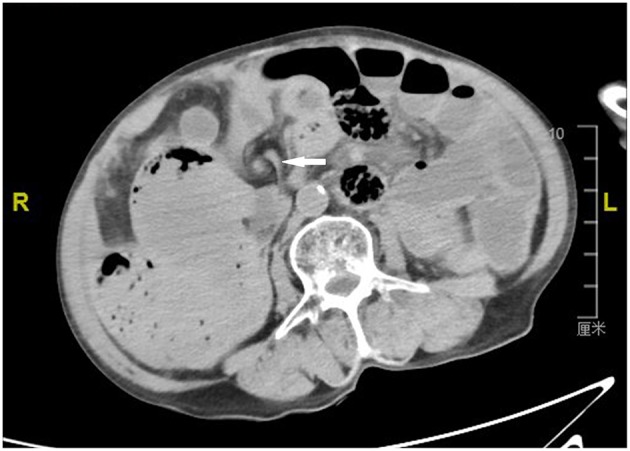
Computerized axial tomographic scan showing the “whirl” of the mesenteric artery and vein (arrow).

**Figure 3:**
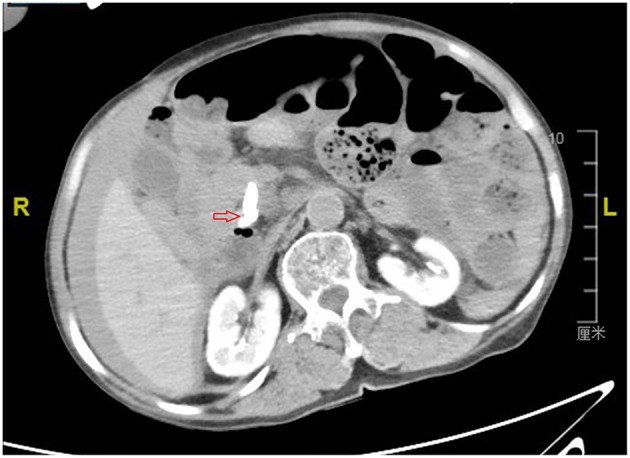
Computerized axial tomographic scan showing the stone of the common bile duct (arrow).

**Figure 4:**
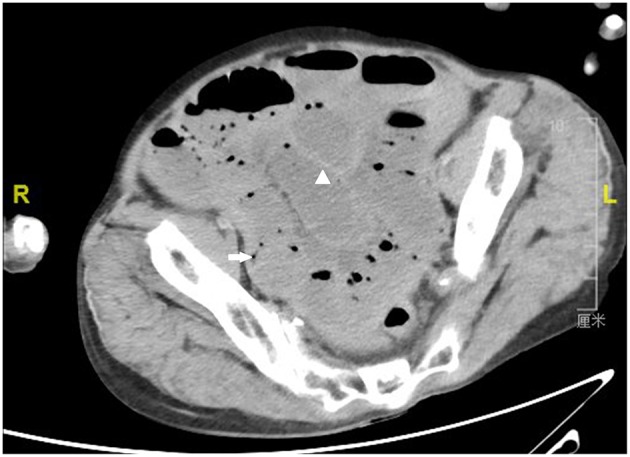
Computerized axial tomographic scan showing the thickening (arrowhead) and bubble (arrow) of the small intestine wall.

By examining the CT image, we could see the original choledochojejunostomy (Figure 1), the “whirl” of the mesenteric artery and vein (Figure 2), the stone of the common bile duct (Figure 3), and the thickening and bubble of the small intestine wall (Figure 4). The complete blood count showed that the leukocyte count was 20.81 × 10^9^/L, hemoglobin was 68 g/L, neutrophil percentage was 82.1%, and platelet count was 149 × 10^9^/L; the renal function test showed that creatinine was 140.22 μmol/L, and the urea was 8.89 mmol/L; Ascites amylase was 236.22 U/L; electrolyte and coagulation function were normal. According to the results of the CT image, combined with the clinical manifestations, past history, physical examination and auxiliary examination, the initial diagnosis was volvulus with necrosis, acute diffuse peritonitis, a common bile duct stone, and diffuse ascites. We gave rapid rehydration and expansion, and actively improved preoperative preparation. After the vital signs were stabilized, an emergency laparotomy was performed.

**Figure 5:**
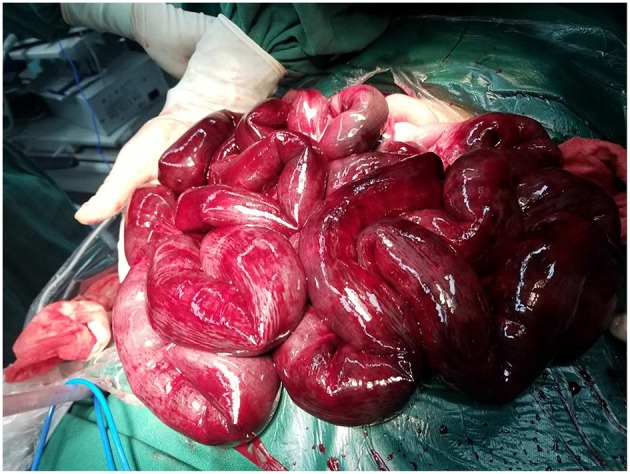
Necrotic, blackened, and not peristaltic intestine canal.

**Figure 6:**
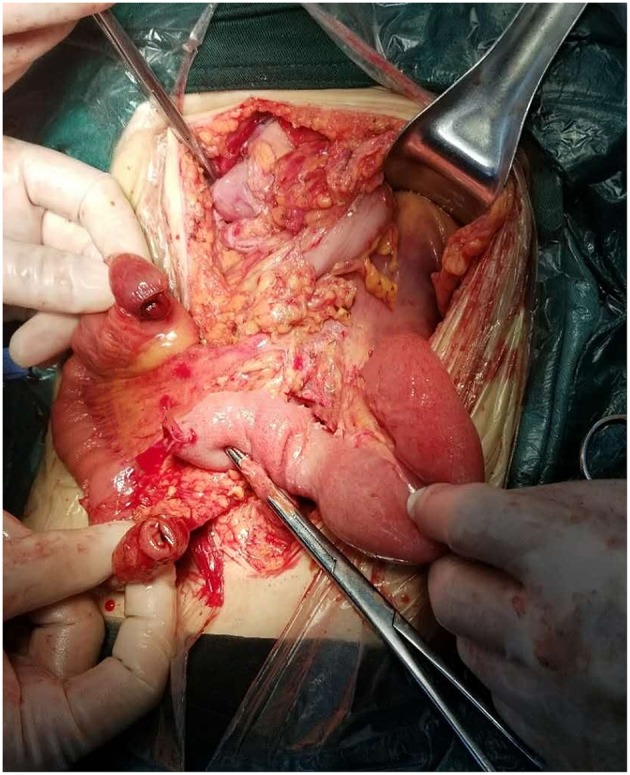
Jejunum of interposition of the original choledochojejunostomy.

**Figure 7:**
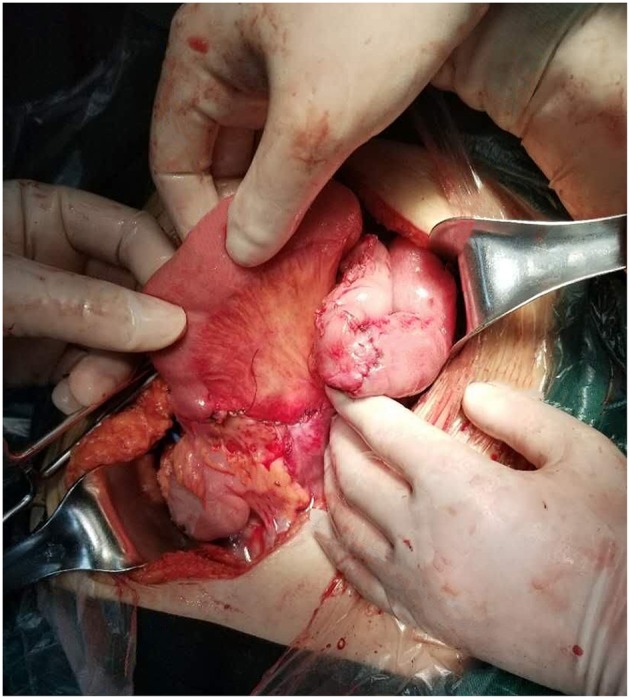
Jejunum of interposition anastomosed between the remnant jejunum and ileocecal junction.

During the operation, ~2,000 mL of odorous dark red effusion was observed in the abdominal cavity. The ileal loop was herniated from the back of the anti-adverse peristaltic loop of the original choledochojejunostomy, and the mesentery was twisted counterclockwise. Near the root of the rotated mesentery, there was an annular adhesive cord band that compressed the twisted mesentery and the intestine. After separating the adhesive cord band, we reduced the torsion of the intestinal tube, which revealed that the output intestinal loop above the ileocecal junction 5 cm and below the choledochojejunostomy 25 cm was necrotic, blackened, not peristaltic, had edema, and there was exudation of the intestinal wall (Figure 5), over a length of ~2.6 m. The blood supply of the input intestinal loop ~30 cm was acceptable, close to the normal state of the intestine. We dissected the intestinal canal from 25 cm below the choledochojejunostomy, 5 cm above the ileocecal junction and the original Roux-en-Y jejunum-to-jejunum anastomosis, and removed the necrotic intestine. In view of the short residual small intestine, we fully dissociated the anti-adverse peristaltic loop of the original choledochojejunostomy in order to avoid a USBS, after which we observed the jejunum of interposition about the length of 25 cm (Figure 6). Therefore, we dissected the intestine canal ~1 cm below the original choledochojejunostomy and anastomosed it between the remnant jejunum and the ileocecal junction (Figure 7); Varus anastomosis was performed on the anterior and posterior intestinal walls and the muscular layer was strengthened.

**Figure 8:**
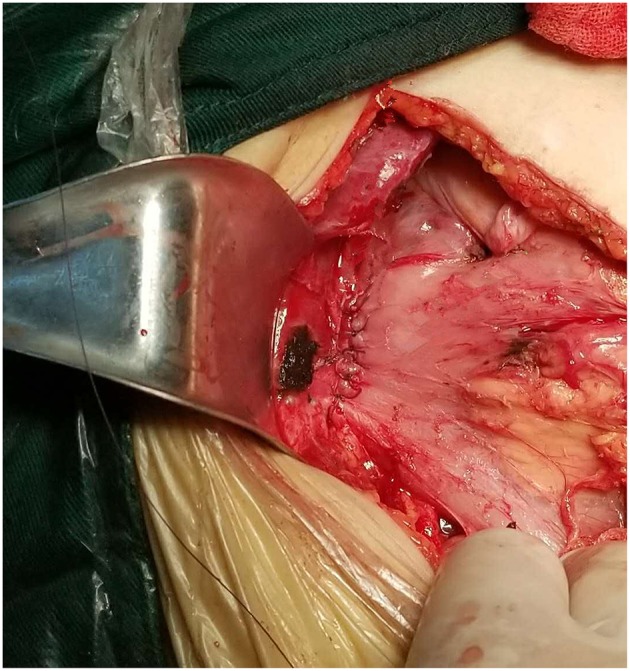
Side-to-side anastomosis of the bile duct and duodenum.

After reconstruction, the small intestine was ~60 cm. Preoperative examination suggested the presence of a common bile duct stone, and intraoperative exploration of the ampullary calculus of the common bile duct was dilated. To reduce the impact of surgical trauma on the body, we decided to perform side-to-side anastomosis of the bile duct and duodenum (Figure 8). We dissected the intestinal wall at the junction between the duodenal bulb and the descending part of the duodenum and anastomosed ~1 cm below the original choledochojejunostomy; Varus anastomosis was performed on the anterior and posterior intestinal walls and the muscular layer of the anterior wall was strengthened. Finally, a gastrostomy was performed to avoid the reflux of gastric contents through the biliary tract due to excessive pressure. The operation time was 2.5 h. After the operation, the patient recovered successfully and was discharged uneventfully 11 days later. During the follow-up of over a year, the patient did not present with diarrhea or other discomfort.

## Discussion

A volvulus is a closed loop intestinal obstruction caused by the compression of both ends of the intestinal loops after the intestinal loop rotates along the long axis of the mesentery, and the mesenteric vessels are blocked. A volvulus obstructs mesenteric blood circulation, potentially leading to necrosis and toxic shock in the short term; it is a common severe acute complication of surgery in the abdomen. The condition is dangerous, requires urgent attention and has a high mortality rate. Therefore, clear diagnosis, accurate positioning, and timely treatment are crucial for the prognosis of patients. Due to non-specific clinical manifestations, CT and multiplanar recombination play an important role in identifying signs of volvulus, such as vascular torsion and transition points (3). With the wide application of multislice spiral CT in clinical practice, Iannelli et al. (4) suggested that a CT plain scan, enhancement, and image post-processing techniques could not only find the general signs of acute intestinal obstruction, but also clearly and directly observe the rotated mesentery and the small intestinal shadow with the center of the rotated mesentery as the axis, which is the typical manifestation of the so-called “whirl sign.”

The “whirl sign” was first discovered by a foreign scholar, Fisher (5), on the CT image of a case of volvulus and described as the vascular image with torsion of the mesenteric artery at its center. Since then, most scholars have supported the “whirl sign” as a specific sign for CT diagnosis of small bowel volvulus (SBV). Meanwhile, some authors believe that the “whirl sign” is not a specific sign of volvulus, which can also be seen in normal variation, postoperative changes, and so on. Blake et al. (6) pointed out that this sign may also occur in patients with malrotation of the small intestine, intestinal adhesion, intestinal tumor, and abdominal and pelvic surgery. Gollub et al. (7) also reported that the “whirl sign” shown by CT imaging did not always suggest twisting of the small intestine. Falk et al. (8) believed that the diagnosis of volvulus requires signs of changes not only in intestinal canal alignment, but also abnormal vascular alignment, because at the same time as the intestine is twisted, the blood vessels in this mesentery must also be twisted. de Korte al. (9) reported a case of “whirl sign” of SBV shown by CT scan, which was confirmed by a laparotomy as the entire small intestine torsion around the superior mesenteric artery. In addition, Tam et al. (10) also reported a case of an emergency CT scan showing a classic “whirl sign,” which was confirmed by emergency laparotomy as a 270° primary SBV of the terminal ileum. The imaging manifestation of “whirl sign” also appeared in this reported case. We believe that the “whirl sign” of the CT image is the key sign of volvulus, and refers to whether the intestinal wall is thickened, edema, and pneumatosis are observed, whether the strengthening of intestinal wall is weakened, whether the intestinal lumen is hydropic, pneumatosis, dilatation, and whether the abdominal cavity has ascites and other signs. These features, combined with the analysis of the condition, are helpful for the correct preoperative diagnosis of volvulus.

Intestinal necrosis on the CT scan is characterized by different degrees of dilatation, hydrops, pneumatosis of the intestinal lumen and pneumoconiosis between the intestines, intestinal wall, and vascularization. Its pathological basis is that insufficient blood supply of the small intestine leads to a series of changes. In the early stage of intestinal ischemia, intestinal peristalsis decreases, and intestinal wall exudation increases. Therefore, the intestinal lumen dilates and accumulates gas, leading to damage to the nerves in the intestinal wall, loss of tension, which result in intestinal lumen dilatation and pneumatosis, excessive proliferation of intestinal bacteria, and pneumatogenic bacteria invading the intestinal wall submucosa and veins. Increased pressure in the intestine lumen and mucosal ulcers allow gas to enter the intestinal wall and veins (11). Wiesner et al. (12) showed that intestinal wall and vascular pneumatosis are highly correlated with full-thickness necrosis of the intestinal wall, which is an irreversible sign of intestinal ischemia and requires immediate surgery.

Studies have shown that the annual incidence of SBV is 1.7–5.7 per 100,000 adults in Western countries and 24–60 per 100,000 adults in Africa, Middle East, and Asia (3, 13, 14). SBV is easily associated with strangulated intestinal obstruction. If there is mesenteric torsion, intestinal necrosis can occur in a short period of time, and it is not treated in time, the mortality rate is relatively high. Klein et al. (15) reported a case of death due to acute hemorrhagic necrosis of the jejunum caused by volvulus. The mortality rate of SBV is estimated to be 9–35%, but it increases to 20–100% with intestinal necrosis (13, 16). The timing of surgical intervention remains an issue. If the patient has signs and symptoms consistent with peritonitis or acute vascular insufficiency, urgent surgical intervention is required; if the patient does not have symptoms and signs of peritonitis or acute abdomen, a non-surgical procedure may be used first. Coe et al. (14) showed that 65.2% of patients with SBV required surgical intervention; in addition, patients who underwent operation had lower mortality (5.94 and 11.65%, respectively) compared with patients who received non-surgical treatment. Therefore, surgery is the main means of treatment (10, 14, 16), in the case of suspected impotence of intestinal necrosis, surgery is urgently needed, and active intestinal tubes should be kept as much as possible during the operation. Acute intestinal torsion and necrosis often lead to large-scale small bowel resection, which results in USBS, the avoidance of which is the key to the success of the operation. In this reported case, the problem of short residual small intestine after excision of necrotic small intestine was considered. Therefore, the method of reanastomosis was performed by interpositioning the jejunum of the original bile duct jejunum Roux-en-Y anastomosis to avoid the occurrence of the USBS, thereby improving the functional outcome of the operation.

## Conclusion

CT is of great value in the analysis of the etiology and obstruction point of intestinal obstructions. Mastering its key diagnostic signs and rigorously analyzing patient symptoms can lead to correct diagnoses and guide the formulation of treatment protocols. Strangulated intestinal obstruction should be diagnosed as soon as possible and surgery should be performed in time. Flexible use of an active intestinal tube during the operation can avoid the occurrence of USBS, thus improving the success of the operation.

## Ethics Statement

The case report was conducted in accordance with the ethical standards set out in the Helsinki Declaration of 1964. The informed consent was obtained from the patient's family. The patient's son gave the consent to include the data in the present case study.

## Author Contributions

All the authors were involved in patient management. ZZ conceived the study. ZZ and CH did the clinical exam and performed the surgery. ZN and CH collected the clinical data and images. CH drafted the manuscript and it has been read and approved by all the authors.

### Conflict of Interest Statement

The authors declare that the research was conducted in the absence of any commercial or financial relationships that could be construed as a potential conflict of interest.
